# Impact of aircraft CO, NO_2_, and SO_2_ emissions on public health in Chinese airports

**DOI:** 10.7189/jogh.16.04210

**Published:** 2026-08-14

**Authors:** Jian-bin Wu, Qiang Cui

**Affiliations:** School of Economics and Management, Southeast University, Nanjing, China

**Keywords:** public health, aircraft, GEMM model, CO, NO_2_, SO_2_

## Abstract

**Background:**

The rapid development of China’s civil aviation industry has resulted in a greater impact of aircraft emissions on the public health of residents living near airports. Despite attention given to aviation-related environmental issues, research on the health effects of aircraft-related CO, NO_2_, and SO_2_ emissions remains limited. We aimed to fill this gap by examining the health impacts of these pollutants on populations surrounding airports in China.

**Methods:**

We collected basic flight takeoff and landing information from the Civil Aviation Administration of China and calculated the NO_2_, CO, and SO_2_ concentrations at each airport using the Gaussian diffusion model. We also calculated the premature deaths caused by NO_2_, CO, and SO_2_ emissions at each airport using the Global Mortality Model and population data near the airports.

**Results:**

In our sample, CO, NO_2_, and SO_2_ emissions were associated with a higher attributable mortality burden in men than in women, and a greater burden is observed among older age groups. Among the three pollutants, CO accounts for the largest number of respiratory disease deaths. Lastly, we observed the highest number of attributable deaths in 2019.

**Conclusions:**

Our findings give new insights into the relationship between aircraft pollutant emissions and health outcomes in China, and thus provides a valuable reference for future research and policy formulation on how aviation impacts public health.

According to the International Energy Agency, China accounts for 27.9% of global CO_2_ emissions, with civil aviation carbon emissions accounting for about 1% of this output, making the country the second largest carbon emitter in the global civil aviation transport sector [1]. By the end of 2023, China’s civil aviation transport passenger volume will have reached 620 million, representing an increase of 146.1% from 2012 [1]. The total transport turnover will reach 118.83 billion ton-kilometres, amounting to a year-on-year increase of 98.3% [1]. Although the pollution from the civil aviation industry is relatively smaller than that of other modes of transportation, it continues to grow rapidly, while emission reduction efforts are hindered by high investment requirements, long research and development cycles, and strict safety requirements [1]. According to the International Civil Aviation Organization, airport-related emissions affect the atmospheric environment, and long-term exposure to harmful substances emitted by aircraft threatens human health [2].

Aircraft activities not only exacerbate climate warming and damage the ozone layer by emitting greenhouse gases and pollutants, but also negatively impact the surrounding environment and human health due to noise pollution [2,3]. Studies have shown that exposure to a variety of air pollutants, especially fine particulate matter, increases the risk of cerebrovascular and neuropsychiatric diseases, and aircraft cruise emissions can even lead to premature death [4]. In addition, aircraft produce a large amount of NO_x_, SO_2_, CO, hydrocarbons, PM_2.5_ and PM_10_, volatile organic compounds, *etc*. during the landing and take-off (LTO) phase, which significantly affects the environment and health around the airport [5,6].

Although CO is not a greenhouse gas, it can produce pollutants such as ozone through chemical reactions, thereby affecting the climate. Reducing traffic-related PM_2.5_ and CO in Tianjin was associated with fewer deaths from cardiovascular and respiratory diseases; cumulative deaths from these two causes decreased by 156 and 961, respectively, from 2016 to 2019 compared with 2015 [7]. Long-term low-concentration CO exposure can cause chronic headaches, cognitive impairment, memory loss, *etc*., while long-term high-concentration exposure can cause permanent heart and nervous system damage [7]. In turn, NO_2_ has been associated with high mortality and morbidity worldwide, with short-term exposure increasing mortality risk, especially for vulnerable populations [8]. Studies have shown that short-term exposure to PM_2.5_, NO_2_, and O_3_ could be associated with asthma mortality [9]. In addition, NO_2_ concentration was shown to be closely related to bronchial symptoms and decreased lung function in asthmatic children, with the effect being more pronounced in high temperature environments [10]. Lastly, as one of the main sources of global air pollution, SO_2_ can aggravate respiratory diseases such as asthma and chronic bronchitis and increase the risk of cardiovascular disease [11,12].

Ats of aircraft emissions, CO, NO_2_, and SO_2_, present as potential hazards to air quality, climate change, and human health. Many studies have used emission factor methods to estimate the concentration of pollutants generated by aircraft activities [13–16]. For example, one study combined emission factors and flight trajectories and used atmospheric diffusion models to simulate pollution distribution around airports [13]. Another used chemical transport models to evaluate the impact of emissions from London Heathrow Airport on local air quality [14]. Elsewhere, a multi-pollutant model was used to distinguish the contribution of vehicle and aircraft emissions near airports to NO_2_ concentrations [15]. One investigation of the emission factors of different LTO stages found that emissions during the take-off stage were significantly higher than those in other stages [16].

Obtaining accurate, up-to-date aviation emission inventories are crucial for analysing the impact of greenhouse gases and air quality around airports. These inventories are usually based on engine certification emission factors, which accurately assess the impact of air quality around airports by considering factors such as fuel flow and engine operating time [17]. Organisations such as NASA and EUROCONTROL have developed the Global Aviation Emissions Inventory and the Aviation Fuel Use and Emissions Inventory System, respectively, to improve the estimation of aviation emissions and provide essential data support for environmental impact and health risk assessments [18,19]. In China, aircraft LTO-stage pollutant emission factors and emission inventories have also been initially established for the Greater Bay Area [20]. The National Earth System Science Data Center has developed the China High Concentration Air Pollutant Dataset to monitor seven near-ground air pollutants, including NO_2_, SO_2_ and CO [20,21]. China started late in airport emission research, has limited public data, and has many emission inventories that still rely on historical data, resulting in inaccurate results [22–24].

The integrated exposure-response model (IER) and the global exposure mortality model (GEMM) are commonly used for assessing the long-term health effects of pollutants [25–27]. The IER model was originally used to assess PM_2.5_ and was later widely used in global disease burden research, while the GEMM model is more adaptable and can handle higher pollution levels and a wider range of population data [28–30]. In one example, a GEMM model was applied to analyse global PM_2.5_ exposure levels, showing that the GEMM model provides a more accurate health risk assessment in highly polluted areas [28]. However, there are very few quantitative studies on the health effects of NO_2_, SO_2_, and CO.

Here, we first adopted a Gaussian diffusion model [31] to calculate the emission concentrations of Chinese airports from January 2006 to December 2023 [32]. Then, we used the average concentration in January 2006 as the threshold and uses the GEMM model to calculate the number of deaths caused by CO, NO_2_, and SO_2_ per month from January 2006 to December 2023.W

## METHODS

### Calculation of CO, SO_2_, and NO_2_ concentration

We first gathered monthly aircraft take-off and landing data from January 2006 to December 2023 from the Civil Aviation Administration of China website (**Table 1**). Using the modified BFFM2-FOA-FPM method (**Table 3**) and considering the annual proportions of wide-body, narrow-body, and regional aircraft (**Table 2**), we calculated the average emissions of CO, SO_2_, and NO_X_ (with NO_2_ accounting for 5%) for each LTO stage of the aircraft, as well as the total monthly emissions.

**Table 1:** China's civil aviation industry monthly take-offs and landings (January 2006 to December 2023)

	Month											
**Year**	1	2	3	4	5	6	7	8	9	10	11	12
2006	118,247	110,504	119,228	120,656	126,539	122,629	136,680	142,489	137,583	137,791	127,312	127,211
2007	121,821	126,292	135,154	130,628	140,190	131,458	146,051	153,390	138,215	140,864	127,610	127,025
2008	116,909	123,332	129,041	139,643	129,528	113,885	132,653	129,884	123,954	128,762	133,850	124,513
2009	128,015	133,218	126,666	137,539	135,855	137,628	141,359	169,414	152,525	160,574	147,892	149,030
2010	141,046	139,605	148,263	154,396	159,285	155,408	175,752	179,242	182,538	177,581	175,591	173,860
2011	189,131	174,735	180,795	189,688	192,010	186,204	202,772	216,218	209,286	213,866	202,353	199,328
2012	195,966	186,345	207,773	207,437	203,235	204,118	229,580	234,412	225,504	223,445	219,202	217,227
2013	212,311	204,328	235,294	229,034	229,496	228,613	251,008	262,983	248,277	248,319	238,235	234,454
2014	242,437	230,672	253,025	253,193	252,689	247,773	277,185	285,840	271,513	280,714	271,429	276,891
2015	272,059	271,176	298,235	288,235	290,756	281,681	310,588	325,966	303,992	317,773	304,916	307,563
2016	320,168	298,319	326,471	326,471	323,109	321,008	350,420	364,706	347,479	359,664	344,118	355,462
2017	363,866	332,773	370,168	365,966	375,630	367,227	389,076	399,160	389,916	402,521	395,798	401,681
2018	394,538	376,471	425,630	421,849	419,328	413,445	435,714	449,580	426,050	443,277	424,370	433,193
2019	446,218	405,462	447,899	441,597	452,941	443,697	476,050	484,454	460,084	469,748	450,420	454,202
2020	424,370	105,882	163,866	165,966	226,471	254,202	289,076	319,748	351,261	364,286	347,899	336,555
2021	266,807	210,924	374,790	386,975	385,714	328,151	361,765	206,303	289,496	307,983	219,748	257,983
2022	272,269	259,244	189,496	123,950	163,445	223,950	285,714	260,504	201,261	178,992	160,924	197,059
2023	310,504	333,193	364,286	391,176	407,983	425,630	477,731	487,815	449,580	462,605	427,311	452,941

**Table 3:** LTO emissions of various gases from different types of aircraft

Year	Wide-body aircraft ratio	Narrow-body aircraft ratio	Regional aircraft ratio
2006	0.178356713	0.70240481	0.119238477
2007	0.1280077	0.787295476	0.084696824

**Table 2:** Percentage of wide-body aircraft, narrow-body aircraft and regional aircraft

Year	Wide-body aircraft ratio	Narrow-body aircraft ratio	Regional aircraft ratio
2006	0.178356713	0.70240481	0.119238477
2007	0.1280077	0.787295476	0.084696824
2008	0.163621922	0.727561557	0.108816521
2009	0.163621922	0.727561557	0.108816521
2010	0.141861938	0.766941102	0.09119696
2011	0.138400454	0.766307431	0.095292116
2012	0.137063655	0.770533881	0.092402464
2013	0.134453782	0.790849673	0.074696545
2014	0.133333333	0.802531646	0.064135021
2015	0.131698113	0.811320755	0.056981132
2016	0.13220339	0.813220339	0.054576271
2017	0.134708738	0.811893204	0.053398058
2018	0.067326189	0.884034075	0.048639736
2019	0.145364065	0.804347826	0.050288109
2020	0.145016654	0.803228286	0.05175506
2021	0.126052858	0.821340747	0.052606394
2022	0.126052858	0.821340747	0.052606394
2023	0.126052858	0.821340747	0.052606394

Next, we calculated the turnover ratio for each airport (average number of take-offs and landings), allowing emissions to be distributed across airports to estimate the LTO emissions for each airport by month. We then averaged the emission flow rate over time by dividing the total emissions by the number of days in the month, hours in a day, and seconds in an hour, resulting in values expressed in g/s. Finally, we employed a Gaussian diffusion model to calculate the monthly average concentrations of CO, SO_2_, and NO_2_ at each airport, expressed in *μ*g/m^3^.

According to the modified BFFM2-FOA-FPM method, the SO_2_ emitted per unit fuel consumption is fixed, that is, 3.87g of SO_2_ is emitted per kilogram of aviation kerosene, so this article calculates the sulfur dioxide databased on the turnover and fuel per unit turnover published by the Civil Aviation Administration of China.

According to the emission flow, the average concentration value is calculated by the Gaussian diffusion model, which has been widely applied in calculating emission concentration [31,33]. The Gaussian diffusion model considers the influence of factors such as wind speed and temperature. Its specific steps are as follows:



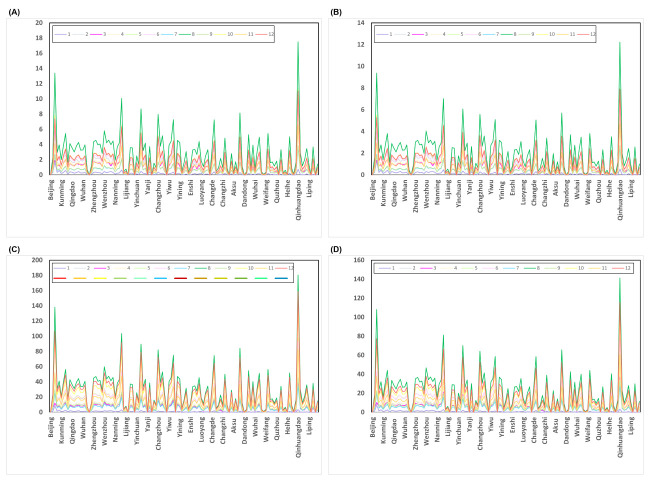





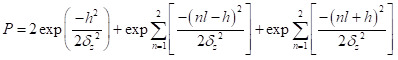



*C* is the concentration (*μ*g/m^3^), E is the emission flow (g/s), *μ* is the wind speed (m/s), this study sets *μ* = 3.7. *δ_y_*, *δ_z_* are the diffusion coefficient of the horizontal and vertical directions, this study sets *δ_y_* = 1.33, *δ_z_* = 1. *h* is the effective height. n is the number of smoke reflections, in this study, *h* = 2 and n = 3. *l* is the mixing layer height (m). Generally, for the coastal airports, *l* = 900, and for the other ones, *l* = 1100. For the possible emission height that may have effects on humans *y*, this study selects *y* = 1, *y* = 2, and *y* = 3, and then calculate the average value as the final concentration.

We employed the Gaussian dispersion model because airport-scale air pollution exposure exhibits significant spatial heterogeneity, and existing monitoring stations have extremely limited coverage around airports, making it difficult to directly provide high-resolution continuous concentration fields. Reconstructing the spatial distribution of pollutants using dispersion models is a standard method in health impact assessments of aviation emissions [34]. This type of model has been used in prior studies to simulate near-field pollution from airports and transportation sources [35]. Its basic assumption is that the emission rate is transformed into spatial concentration distribution by parameterising the atmospheric turbulent diffusion process. In the context of airport low-altitude emissions (LTO cycle), it is widely used for near-field exposure assessment of pollutants such as NO_2_, CO, and SO_2_, and has been applied in health risk studies at airports in multiple countries. Its rationality has been systematically validated in the literature.

Our analysis focuses not on the precise reconstruction of absolute concentrations, but rather on the relative differences in exposure based on spatial distribution and their attribution for health effects. In this type of health burden study, the role of diffusion models is to provide a high-resolution exposure field, and their uncertainties are typically controlled by subsequent exposure-response functions and uncertainty propagation analysis. We also do not assume that air pollution in airport areas originates solely from aviation activities. In reality, airports and their surrounding environments are influenced by multiple emission sources, including road traffic, auxiliary power units, ground support equipment, nearby industrial activities, and urban background pollution. Accordingly, our aim is not to achieve absolute source attribution, but rather to estimate the incremental contribution of aviation-related emissions using an emissions inventory coupled with a dispersion modelling framework. The concentrations reported here, therefore, do not represent total ambient pollution levels at airports. Instead, they reflect the marginal exposure attributable to aviation activities, derived using standard source apportionment approaches. This methodology is consistent with established practices in the airport health impact assessment literature [31]. To summarise, in complex, multi-source urban environments, precise single-source attribution is rarely achievable based on observational data alone. Consistent with common principles in environmental health research, we adopted a ‘source inventory + dispersion modelling + scenario analysis’ framework to estimate the marginal health impacts of specific emission sources, rather than attempting to fully explain observed pollutant concentrations.

### GEMM model

The GEMM functions in two steps: calculating relative risks (RRs) by comparing actual and threshold concentrations and estimating premature deaths using data on population size and baseline mortality rates [36]. This model, which has been widely used in health impact assessments [37-39], predicts hazard ratios for RRs in logarithmic form, extending beyond the observed exposure range, with changes decreasing as exposure increases:



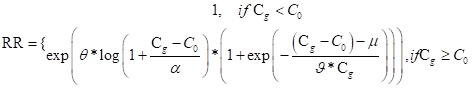



*C_g_* is the actual concentration (*μ*g/m^3^) and *C_o_* is the threshold concentration (*μ*g/m^3^). Based on existing literature [36], we set *C*_0_ = 5. *θ*, *α*, *μ*, and *ϑ* are the parameters that need to be set in advance. According to the results of parameter sensitivity analysis [26], we set *θ* at 0.143, *α* at 1.6, *μ* at 15.5, and *ϑ* at 36.8.

Importantly, the GEMM is fundamentally a unified modelling framework built on epidemiological exposure–response functions [26]. Its essence is not limited to PM_2.5_, but to mapping pollutant exposure levels to health outcomes, typically mortality risk. Although the original GEMM was developed using PM_2.5_ cohort data, its underlying philosophy is consistent with the IER model, as both rely on a unified nonlinear risk function framework.

In this study, extending the framework to CO, NO_2_, and SO_2_ does not involve directly applying PM_2.5_ parameters, but is supported by two methodological foundations [40,41]. First, independent epidemiological evidence provides pollutant-specific exposure–response relationships. Projects such as World Health Organization (WHO) HRAPIE and multinational cohort studies offer concentration–response functions or relative risk estimates for these pollutants, forming a basis for constructing tailored exposure–response functions. Second, a continuous risk function consistent with GEMM is adopted, rather than threshold or linear cutoff models, ensuring comparability and additivity of health effects across pollutants within a unified framework. This enables a coherent assessment of multi-pollutant health burdens and aligns with established practices in the literature.

 Concentration threshold settings for air pollutants vary substantially across countries and regions. For NO_2_, the World Health Organization recommends that the one-hour average concentration should not exceed 200 *μ*g/m^3^ to protect public health [42]. Similarly, the European Union sets a one-hour threshold of 200 *μ*g/m^3^, while additionally specifying an annual average limit of 40 *μ*g/m^3^ [43].

For carbon monoxide (CO), the WHO recommends an eight-hour average concentration limit of 9 mg/m^3^ and a one-hour limit of 10 mg/m^3^ [42]. Consistently, the US Environmental Protection Agency establishes a national standard with an eight-hour average threshold of 9 mg/m^3^ [44]. For sulfur dioxide (SO^2^), WHO guidelines suggest that the 24-hour average concentration should not exceed 20 *μ*g/m^3^, and the one-hour average should remain below 500 *μ*g/m^3^ [42]. In contrast, the EU defines a one-hour threshold of 350 *μ*g/m^3^ and a 24-hour threshold of 125 *μ*g/m^3^ [45]. These discrepancies highlight the lack of global uniformity in air quality standards and complicate cross-regional comparisons.

To address this inconsistency, we adopted January 2006 as a unified reference point and evaluated mortality changes relative to that baseline. Rather than relying on externally defined thresholds, this approach standardises the analytical framework for CO, NO_2_, and SO_2_, thereby enhancing the comparability of results. Following a screening process, we selected 126 airports as representative of national-level aviation systems. We used the average pollutant concentrations observed in January 2006 as baseline levels, and applied the GEMM framework to estimate monthly mortality from January 2006 to December 2023, disaggregated by gender and age.

In environmental epidemiology, three main approaches are typically used to define counterfactual exposure baselines: the theoretical minimum risk exposure level; low-end distributional benchmarks (*e.g.* the 5th percentile); and the average conditions during a stable historical period. We adopted the third approach in our analysis, as the use of theoretical minimum risk exposure level or low-percentile thresholds would have introduced inconsistencies across pollutants and regions due to the substantial spatial imbalance in long-term observational data for CO, NO_2_, and SO_2_ across global airports. In contrast, selecting a common historical reference point enables the construction of a structurally consistent baseline across all airports and pollutants, thereby preserving the additivity and spatial comparability of multi-pollutant health impacts.

We selected January 2006 as the baseline based on three considerations. First, it represents a relatively stable phase in global aviation activity, prior to subsequent rapid growth and major policy interventions, and can therefore be viewed as a ‘low-disturbance’ reference state [46]. Second, data coverage for all airports and pollutants was complete during this period, minimising bias from missing observations. Third, we did not interpret the baseline concentration as a health-based safety threshold.

Health effect estimation remains strictly grounded in the continuous exposure–response relationships defined within the GEMM framework. Accordingly, deviations from the baseline, whether increases or decreases, are interpreted as changes in excess risk associated with relative exposure increments, rather than absolute risk thresholds.

We used the following equation for the number of premature deaths:



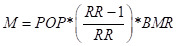



Here, *RR* is the relative risk, *POP* is the population, *BMR* is the base mortality rate, and *M* is the number of premature deaths.

We obtained the base mortality rate of various diseases from the Global Burden of Disease (GBD) dataset [47], and the population data within 50 km of the airport from the LandScan dataset [48]. Given the lack of high-resolution mortality data at the airport scale, we adopted a simplifying assumption that disease-specific mortality rates are spatially uniform at the national level during the study period. Under this assumption, we used age- and sex-specific national mortality estimates from the GBD as baseline values, which we then spatially allocated according to population distribution. Otherwise, we retained population size data and its share of the national total within the study area (defined as a 50 km radius around each airport) from the LandScan dataset. We subsequently applied these population proportions to the national-level, age- and sex-specific mortality totals from the GBD to estimate baseline mortality for populations residing near airports. This approach implicitly assumes that baseline mortality risk *per capita* does not vary systematically across regions. In terms of temporal resolution, as GBD mortality data are primarily reported on an annual basis, so we assumed that baseline mortality is evenly distributed throughout the year. Annual mortality rates are therefore converted into temporally uniform estimates to align with pollution exposure data at monthly or finer time scales.

The effects of factors such as wind speed, humidity and temperature on concentration have already been considered in the concentration calculation process [31], so we did not repeatedly consider them when calculating health effects. Furthermore, we defined a 50 km population buffer centred on each airport to represent the population potentially affected by aviation-related emissions. This spatial scale is selected based on two considerations. First, a prior study has indicated that emissions associated with airport operations – particularly during take-off and landing, as well as the formation of secondary pollutants – can influence air quality over distances of several tens of kilometres [49]. Second, a 50 km buffer ensures adequate population coverage while avoiding statistical instability that may arise from overly small spatial units. As such, this choice reflects a balance between spatial representativeness and statistical robustness. 

To address spatial overlap between adjacent airport buffer zones, we applied a population deduplication (or distance-/weight-based allocation) procedure to avoid double counting. Specifically, when a population grid cell falls within multiple buffer zones, it is either counted only once or allocated based on proximity (*e.g.* nearest-airport assignment). This ensures national-level population consistency and prevents systematic overestimation of attributable mortality.

## RESULTS

### Temporal trends in CO concentrations and CO-attributable mortality

**Figure 1:**
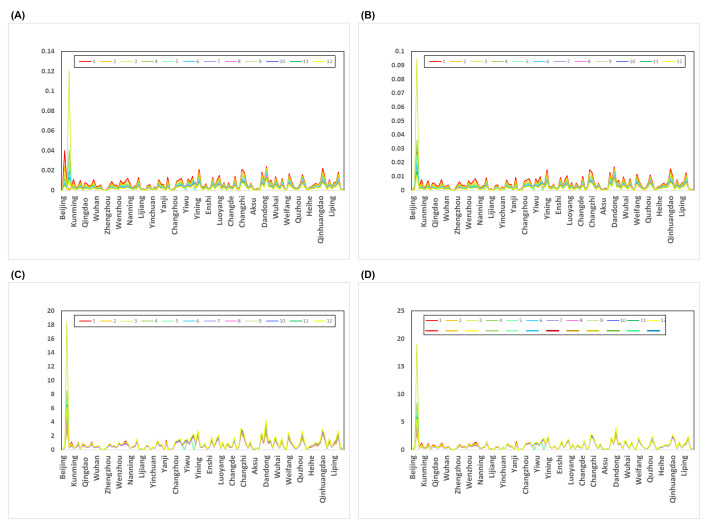
Deaths from cardiovascular disease due to changes in CO concentration. Increase in cardiovascular deaths in men (**Panel A**) and women (**Panel B**) younger than 5 years of age per unit increase in CO concentration at each airport in 2023. Number of cardiovascular deaths in men (**Panel C**) and women (**Panel D**) aged 80-84 years due to CO at each airport in 2023.

From 2006 to 2019, CO concentrations at Chinese airports increased continuously. Concentrations then declined substantially during 2020-2022 and reached a minimum of 34,857.46 *μ*g/m^3^ in 2022. In 2023, CO concentrations rebounded by 33,072.62 *μ*g/m^3^ relative to 2022, representing a 94.9% increase. CO-attributable mortality was highest among men aged 75–79 and women aged 80–84 years. The largest numbers of deaths were observed in winter and early spring, with peaks in February and March 2006 and the fewest deaths in October and December. In 2023, monthly deaths peaked in November and December among men and in January among women (**Figure 1**).

### Temporal trends in NO_2_ concentrations and NO_2_-attributable mortality

**Figure 2:**
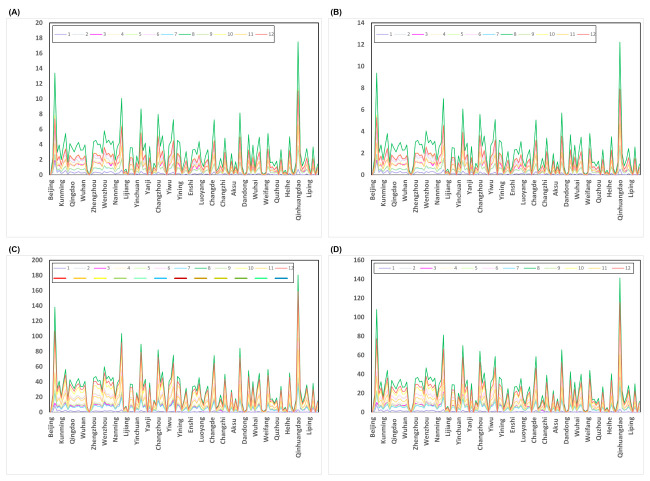
Deaths from respiratory diseases due to changes in NO_2_ concentrations. Increase in respiratory disease deaths per unit increase in NO_2_ concentration at airports in 2023, in men (**Panel A**) and women (**Panel B**) younger than 5 years, and men (**Panel C**) and women (**Panel D**) aged 80-84 years.

From 2006 to 2023, NO_2_ concentrations at airports followed a pattern similar to that of CO and reached their highest annual level in 2019. Between 2019 and 2022, NO_2_ concentrations decreased from 4744.27 *μ*g/m^3^ to 1902.99 ug/m^3^ and then increased to 3708.54 *μ*g/m^3^ in 2023. The highest numbers of respiratory disease deaths attributable to NO_2_ were observed in summer, with a peak in August (**Figure 2**).

### Temporal trends in SO_2_ concentrations and SO_2_-attributable mortality

**Figure 3:**
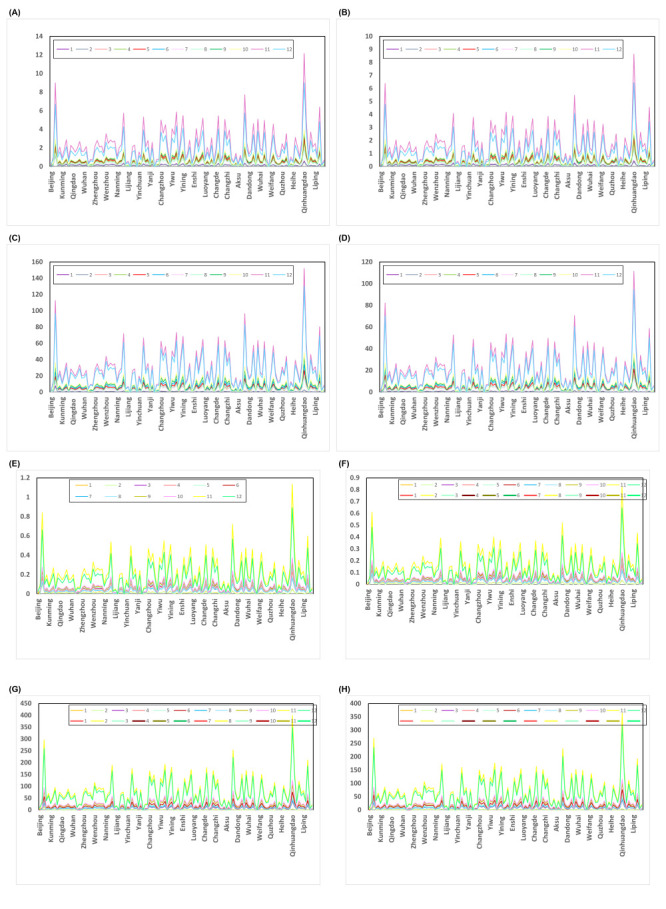
Deaths from respiratory diseases and cardiovascular diseases due to changes in SO_2_ concentrations. Increase in respiratory disease deaths per unit increase in SO_2_ concentration at each airport in 2023, in men (**Panel A**) and women (**Panel B**) younger than 5 years, and men (**Panel C**) and women (**Panel D**) aged 80-84 years. Increase in cardiovascular disease deaths per unit increase in SO_2_ concentration at airports in 2023, in men (**Panel E**) and women (**Panel F**) younger than 5 years, and men (**Panel G**) and women (**Panel H**) aged 80-84 years.

SO_2_ concentrations at airports also peaked in 2019 at 16,010.34 *μ*g/m^3^ and then decreased to 6949.63 *μ*g/m^3^ in 2022, close to the 2009 level. In 2023, the estimated change in deaths was negative in some months relative to the January 2006 baseline. SO_2_-attributable respiratory deaths were highest in November, December, and June, and the mortality burden was greatest in older age groups, particularly men. For deaths associated with unit increases in SO_2_ concentration in 2006 and 2023, the largest increases were observed in November and December. Among adults aged 80–84, the number of attributable deaths was particularly high, and among those aged >85 years, women had higher mortality than men (**Figure 3**).

### Airport-level distribution of pollutant burden in 2023

Across airports, Guangzhou Baiyun, Shanghai Pudong, Beijing Capital, Shenzhen Baoan, and Chengdu Tianfu ranked as the top five for CO, NO_2_, and SO_2_ burden in China (Figure S1 in the **Online Supplementary Document**). In 2023, Guangzhou Baiyun recorded the highest pollutant concentrations, with CO, NO_2_, and SO_2_ concentrations of 3231.19 *μ*g/m^3^, 176.40 *μ*g/m^3^, and 667.48 *μ*g/m^3^, respectively. At Shanghai Pudong, the corresponding concentrations were 2476.81 *μ*g/m^3^, 135.22 *μ*g/m^3^, and 511.64 *μ*g/m^3^, and at Beijing Capital they were 1910.28 *μ*g/m^3^, 104.29 *μ*g/m^3^, and 394.61 *μ*g/m^3^. In 2023, Shanghai Pudong recorded six CO-attributable cardiovascular deaths, compared with two at Guangzhou Baiyun.

### Age- and sex-specific patterns of attributable mortality

From 2006 to 2023, CO-attributable cardiovascular deaths were highest among women aged 80-84 and men aged 75–79, whereas the lowest burden was observed among children aged 5–9. Cardiovascular deaths attributable to CO were higher in women aged >85 years than in men of the same age group (Figure S2, Panels A and B in the **Online Supplementary Document**). The number of attributable deaths declined after age 80 years in men and after age 85 years in women. The largest annual increase in CO-attributable cardiovascular deaths occurred in 2019, with 156,518 additional deaths in men and 115,610 in women.

From 2006 to 2023, NO_2_-attributable respiratory deaths varied across age groups and were highest in 2019, particularly among individuals aged 80–84. Men were generally more affected than women, although in 2023 women aged >90 years showed a greater increase in mortality than men (Figure S2, Panel B in the **Online Supplementary Document**). Attributable mortality was relatively low among individuals aged ≥90 years, whereas the attributable burden was higher among children aged <5 years.

SO_2_-attributable deaths were observed for both respiratory and cardiovascular diseases, with a larger number of attributable deaths recorded for cardiovascular disease. The attributable mortality burden was highest in older age groups, with peaks among men aged 75–79 and women aged 80–84. In contrast, respiratory deaths attributable to SO_2_ were more concentrated among children aged <5 years. The highest numbers of attributable deaths were observed in 2019, followed by 2018. At very old ages, females showed a higher attributable mortality burden than males for respiratory disease (*e.g.* ≥90 years) and cardiovascular disease (*e.g.* ≥95 years) (Figure S2, Panels E–L in the **Online Supplementary Document**).

## DISCUSSION

Our analysis covered 126 airports which were intended to represent national-level airport activity. Based on estimated emissions of CO, SO_2_, and NO_2_ from 2006 to 2023, we conducted a modelling-based assessment to evaluate the potential health impacts associated with aviation-related air pollution. Our results are thus derived from an integrated framework combining emission inventories, dispersion modelling, and transferred exposure–response functions, and therefore represent estimated health risks under specific modelling assumptions, rather than directly observed or causal mortality outcomes [34,49–51].

The temporal analysis suggests that modelled concentrations of all three pollutants peaked around 2019, with an overall increase from 2016 to 2019 after an earlier decline. The year 2019 also corresponded to the largest modelled increases in mortality across pollutants. This pattern is plausible because airport operations can measurably elevate near-surface pollutant concentrations in surrounding communities, while aviation-related impacts may extend beyond the immediate runway environment [51,52].

The estimated health impacts were generally higher in males than in females across most scenarios. Within the model results, CO was associated with relatively larger estimated impacts on respiratory mortality than SO_2_ and NO_2_, while NO_2_ showed smaller effects in this category. By age, the modelled impacts of CO on cardiovascular mortality were concentrated among older populations, with the highest estimates observed among females aged 80–84 and males aged 75–79, and the lowest impacts among children aged 5–9 years. For SO_2_ and NO_2_, the modelled respiratory impacts were also concentrated in vulnerable age groups, with NO_2_ showing relatively larger estimated effects among those aged 80–84 and children under 5 years of age. For SO_2_-related cardiovascular mortality, the highest impacts were observed among males aged 75–79 and females aged 80–84, while younger populations were less affected. Such sex-related heterogeneity is consistent with prior calls to incorporate sex and gender as effect modifiers in air-pollution epidemiology, although the direction and magnitude of the differences vary across studies [53–55].

Seasonal variation was also evident, with stronger CO-related mortality increments in winter and early spring. From a temporal perspective, the largest modelled increases in respiratory mortality for NO2 occurred in August, the strongest CO-related increases in cardiovascular and cerebrovascular mortality occurred from November to January, while SO_2_ showed the largest cardiovascular effects in November and December. Seasonal effect modification has been reported previously in time-series studies of outdoor air pollution and mortality, likely reflecting season-dependent meteorology and exposure patterns [54]. These results are broadly compatible with the wider literature showing positive associations between NO2, SO2, and CO exposure and mortality outcomes. For CO specifically, a nationwide time-series analysis in 272 Chinese cities reported increased cardiovascular mortality per 1 mg/m^3^ increase and no clear threshold [56]. while recent systematic reviews and meta-analyses support pollutant-specific concentration-response functions for NO_2_ and SO_2_ [57,58].

Our provides a nationwide, airport-centred assessment of the health burden associated with aircraft-related emissions of CO, SO_2_, and NO_2_ by integrating modelled pollutant concentrations, population distribution, and concentration-response relationships, thereby offering a consistent basis for comparing relative health burdens across regions and population groups [34,50]. Rather than establishing new causal relationships, the analysis applies an existing exposure–response framework to quantify relative health burden around airports. The baseline concentration is best understood as a reference condition rather than a health-based safety threshold. More broadly, burden of disease studies typically rely on established concentration-response functions and integrated risk models, such as IER and GEMM, because they provide a coherent way to translate exposure into health risk across concentration ranges [26,27,40].

Several limitations should be acknowledged. The analysis depends on modelled concentrations and downscaled mortality rates, so uncertainties in emission inventories, dispersion parameters, population allocation, and spatial resolution remain. Source attribution is also imperfect in densely populated regions with multiple overlapping emissions. In addition, the generalised concentration-response functions introduce uncertainty, particularly for gaseous pollutants, which remain less extensively characterised than PM_2.5_, although the current evidence base does support quantitative burden estimation [56–58]. Future work should incorporate higher-resolution exposure estimates, sensitivity analyses of key model parameters, and observational health data where available.

## Additional material


Online Supplementary Document

## Data Availability

**Data availability:** The datasets generated and/or analysed in this study are available from the authors upon reasonable request.
