# Supplementary material for: Impact of aircraft CO, NO2, and SO2 emissions on public health in Chinese airports
Source: J Glob Health. 2026 Aug 14;16:04210. doi: 10.7189/jogh.16.04210 (PMC13474068; doi:10.7189/jogh.16.04210)
Supplement: Supplementary file 1 [file jogh-16-04210-s001.pdf]

Supplement to: Wu J, Cui Q. Impact of aircraft CO, NO2, and SO2 emissions on public health in Chinese airports. J Glob Health. 2026;16:04210.

This supplementary material contains two parts: Population and disease data and Matlab code of GEMM method.

1. Population and disease data

In this study, the base mortality rate of various diseases is from the Global Burden of Disease (GBD) dataset (2006-2021), and the population data within 50 kilometers of the airport is from the LandScan.

This paper calculates the proportion of the population within 50 kilometers of each airport to the national population (Appendix Table S1), and assumes that this proportion remains unchanged, thereby calculating the number of deaths caused by each disease, gender, age group, and year at each airport under various scenarios.

Appendix S1 Population within 50 km of each airport

Airports	Population rate
Beijing/Capital	0.005441
Shanghai/Pudong	0.016081
Guangzhou	0.004991
Shanghai/Hongqiao	0.016081
Shenzhen	0.005585
Chengdu/Shuangliu	0.006976
Kunming	0.0034
Hangzhou	0.006192
Xi'an	0.007769
Chongqing/Jiangbei	0.002558
Xiamen	0.005295
Qingdao	0.004366
Haikou	0.003602
Changsha	0.004818
Dalian	0.004954
Nanjing	0.004121
Wuhan	0.004084
Shenyang	0.00427
Urumqi	0.000786
Guilin	0.001933
Sanya	0.001269
Zhengzhou	0.00472
Fuzhou	0.004604

Guiyang	0.004153
Jinan	0.004184
Harbin	0.002822
Wenzhou	0.005371
Ningbo	0.003901
Taiyuan	0.004559
Tianjin	0.004689
Nanchang	0.00405
Nanning	0.001797
Changchun	0.003207
Lanzhou	0.003627
Hefei	0.008762
Xishuang Banna	0.000357
Lijiang	0.000622
Zhangjiajie	0.000887
Hohhot	0.003019
Yantai	0.002816
Jiuzhai	0.001626
Yinchuan	0.001889
Lhasa	0.00095
Wuxi	0.006231
Zhuhai	0.002765
Xining	0.002869
Yanji	0.00128
Shijiazhuang	0.004385
Wuyishan	0.00115
Yichang	0.00142
Kashi	0.000622
Changzhou	0.005254
Baotou	0.002805
Weihai	0.003597
Dali	0.001561
Huangshan	0.000898
Yiwu	0.002903
Zhoushan	0.002902
Zhanjiang	0.004777
Diqing	0.001613
Xuzhou	0.002961
Yining	0.004077
Dunhuang	0.000673
Xichang	0.001026
Yibin	0.001982
Korla	0.000272

Enshi	0.000852
Beihai	0.002804
Linyi	0.002224
Liuzhou	0.002275
Lianyungang	0.003458
Luoyang	0.003425
Mudanjiang	0.001401
Mianyang	0.003373
Yuncheng	0.002177
Lincang	0.000525
Changde	0.002489
Nantong	0.005254
Yulin	0.000359
Baoshan	0.000794
Jingdezhen	0.003556
Changzhi	0.002565
Luzhou	0.002961
Hotan	0.000618
Manchuria	0.001011
Xiangyang	0.00227
Aksu	0.000172
Tongren	0.001049
Wanzhou	0.000176
Ganzhou	0.005291
Zhaotong	0.001617
Dandong	0.003791
Qiqihar	0.001338
Nanchong	0.003285
Tongliao	0.000617
Jiamusi	0.003137
Wuhai	0.001293
Nanyang	0.00247
Yancheng	0.003274
Yanan	0.000557
Xilinhot	5.18E-05
Weifang	0.002186
Datong	0.003256
Xingyi	0.001207
Dongying	0.001079
Chifeng	0.000771
Quzhou	0.001499
Jinzhou	0.002467
Dazhou	0.002229

Karamay	0.000158
Jiayuguan	0.000626
Heihe	0.000581
Ulanhot	0.003156
Meizhou	0.001323
Golmud	0.00087
Ji'an	0.001748
Qinhuangdao	0.010232
Kuqa	0.002951
Wenshan	0.000708
Linzhi	0.002195
Tacheng	0.00219
Liping	0.00148
Liancheng	0.001438
Anqing	0.003772
Baise	0.000403
Wuzhou	0.001466

2. Matlab code of GEMM method

The detailed MATLAB code of the GEMM model.

```
CC=[ ];% The threshold of the two diseases
nongdu=[ ];%The detailed concentration of different airports in different years
[n,m]=size(nongdu);
POP=[ ];%The population proportion
BMR1=[ ];% The deaths of the first disease—cardiovascular diseases
BMR2=[];% The deaths of the second disease—respiratory diseases
alpha=1.6;
miu=15.5;
theta=0.1430;
v=36.8;
t=10;% This petameter is for the different age groups. 1:<5;2:5-9;3:10-14; 4:15-19; 5:20-24; 6: 25-29; 7: 30-34; 8:35-39; 9: 40-44; 10: 45-49; 11: 50-54; 12: 55-59; 13: 60-64; 14: 65-69; 15: 70-74; 16: 75-79; 17: 80-84; 18: 85-89; 19；90-94； 20：95+
[n,m]=size(nongdu);
for i=1:n
    for j=1:m
        if nongdu(i,j)<CC(1)
            RR1(i,j)=1;
        else
            z(i,j)=nongdu(i,j)-CC(1);
            RR1(i,j)=exp(theta*log(1+z(i,j)/alpha)*(1/(1+exp(-(z(i,j)-miu)/v/nongdu(i,j)))));
        end
    end
```

```
end% The relative risk of the first disease
for i=1:n
    for j=1:m
        PAF1(i,j)=(RR1(i,j)-1)/RR1(i,j);
        GEMM1(i,j)=BMR1(t,j)*PAF1(i,j)*POP(i);
    end
end% The deaths of the first disease
for i=1:n
    for j=1:m
        if nongdu(i,j)<CC(2)
            RR2(i,j)=1;
        else
            z(i,j)=nongdu(i,j)-CC(2);
            RR2(i,j)=exp(theta*log(1+z(i,j)/alpha)*(1/(1+exp(-(z(i,j)-miu)/v/nongdu(i,j)))));
        end
    end
end% The relative risk of the second disease
for i=1:n
    for j=1:m
        PAF2(i,j)=(RR2(i,j)-1)/RR2(i,j);
        GEMM2(i,j)=BMR2(t,j)*PAF2(i,j)*POP(i);
    end
end% The deaths of the second disease
```

3. Supplementary figures

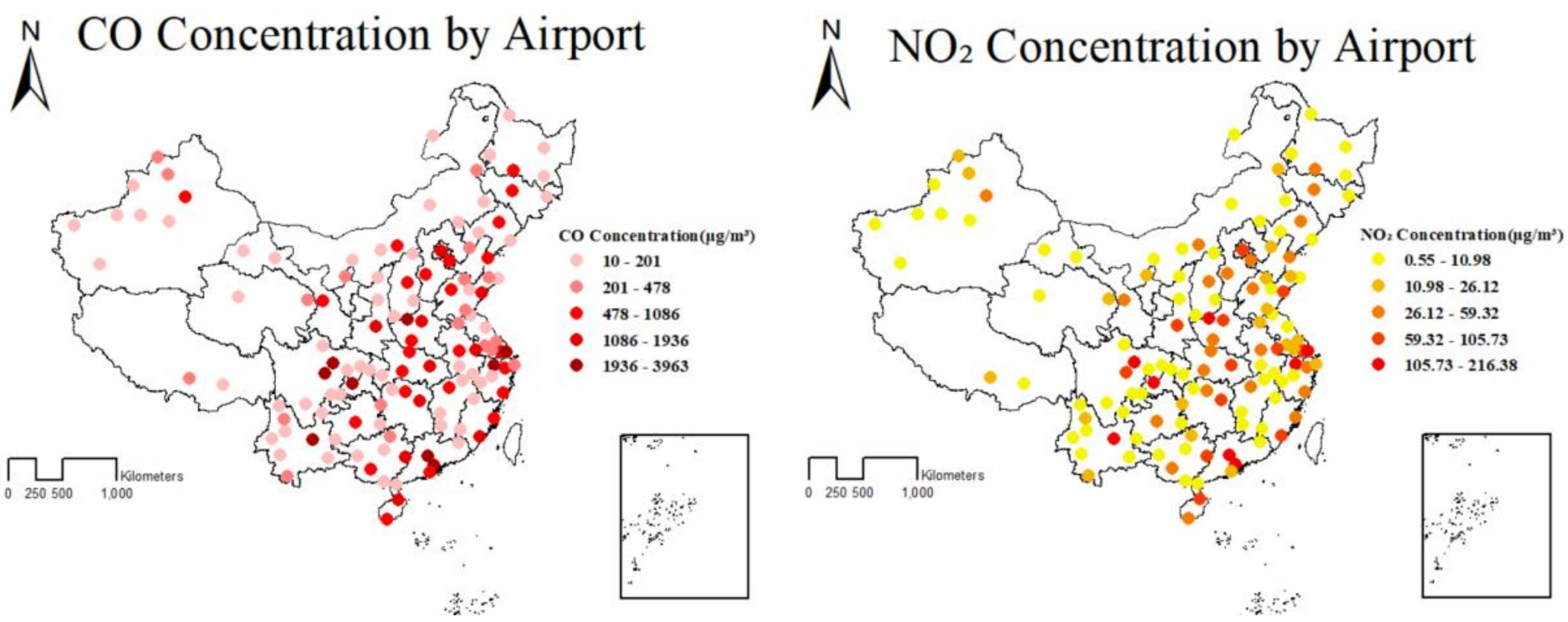

(a) (b)

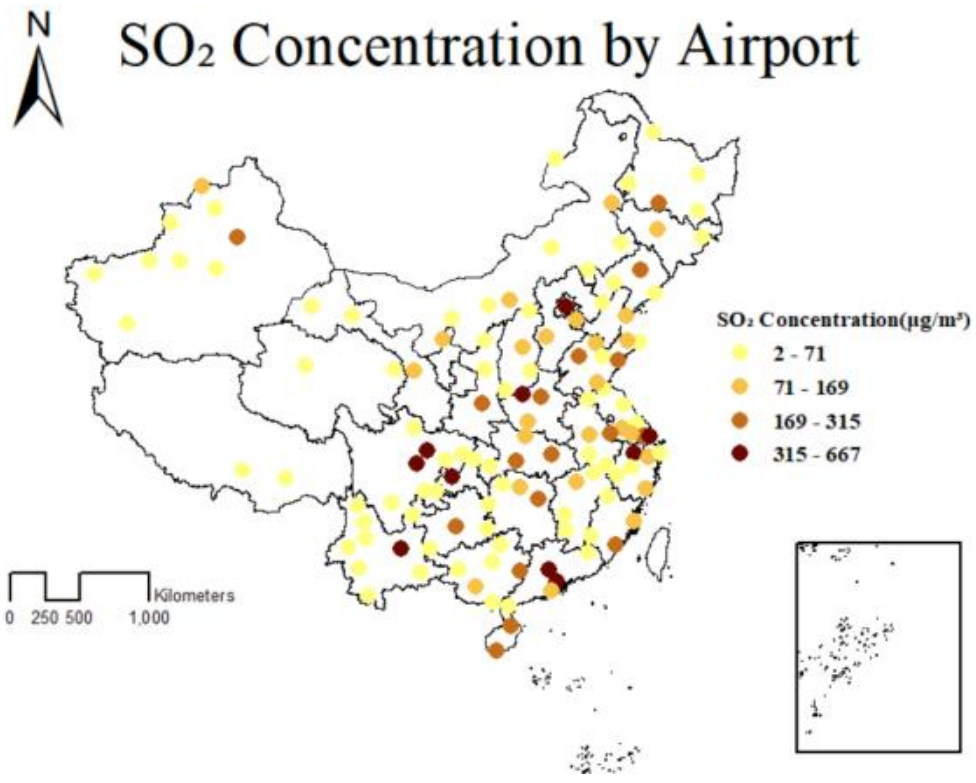

(c)

Figure S1 Concentration of CO,NO_2,SO_2 emissions from 126 airports in China. (a) Concentration of CO emissions from 126 airports in China; (b) Concentration of NO_2 emissions from 126 airports in China; (c) Concentration of SO_2 emissions from 126 airports in China.

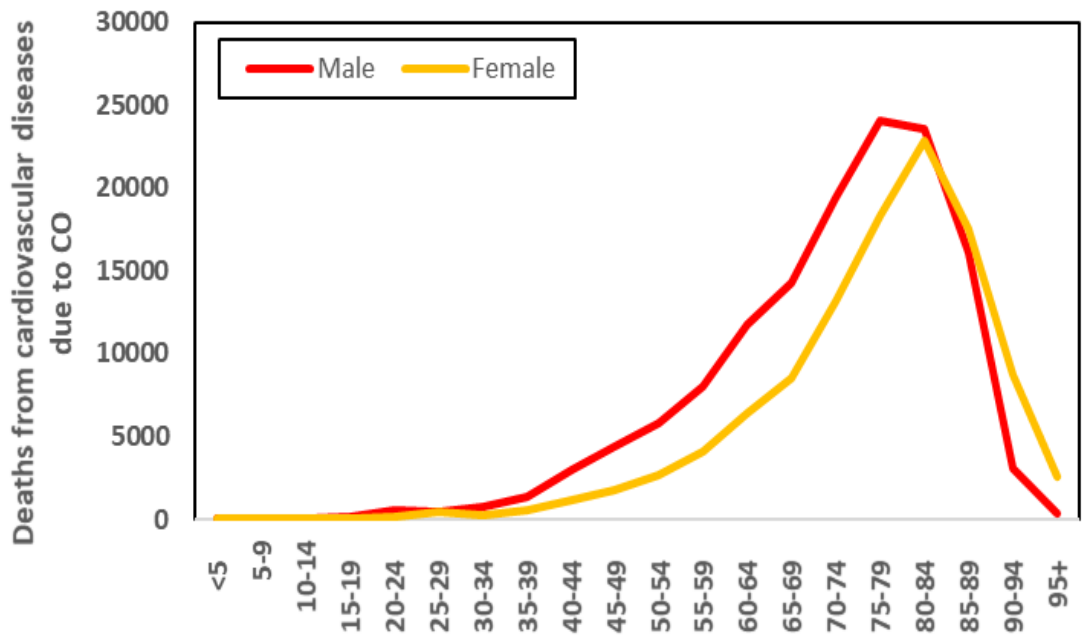

(a)

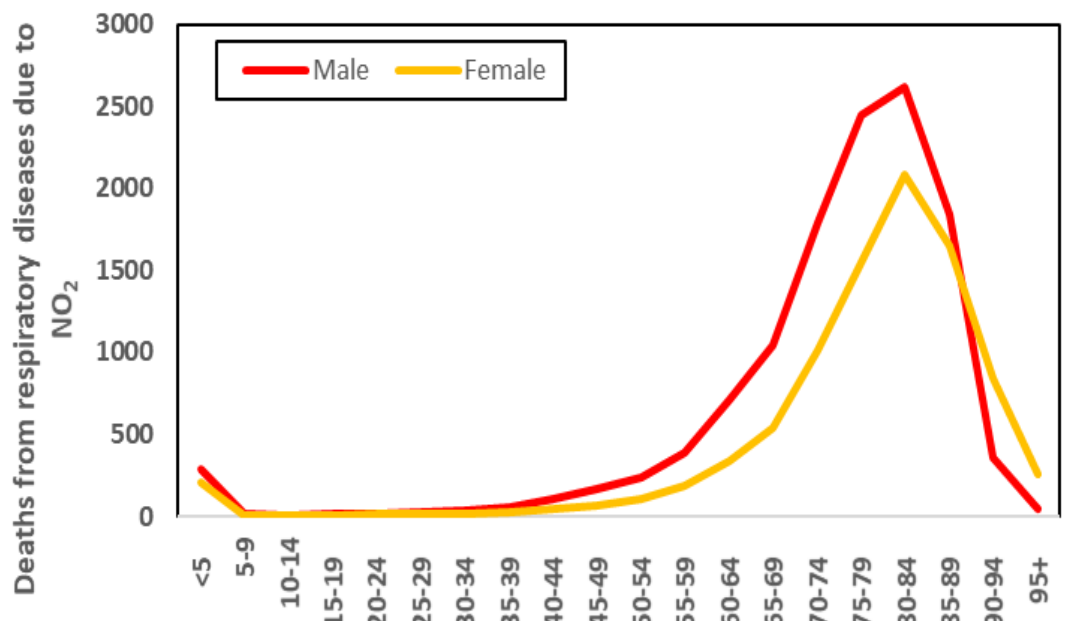

(b)

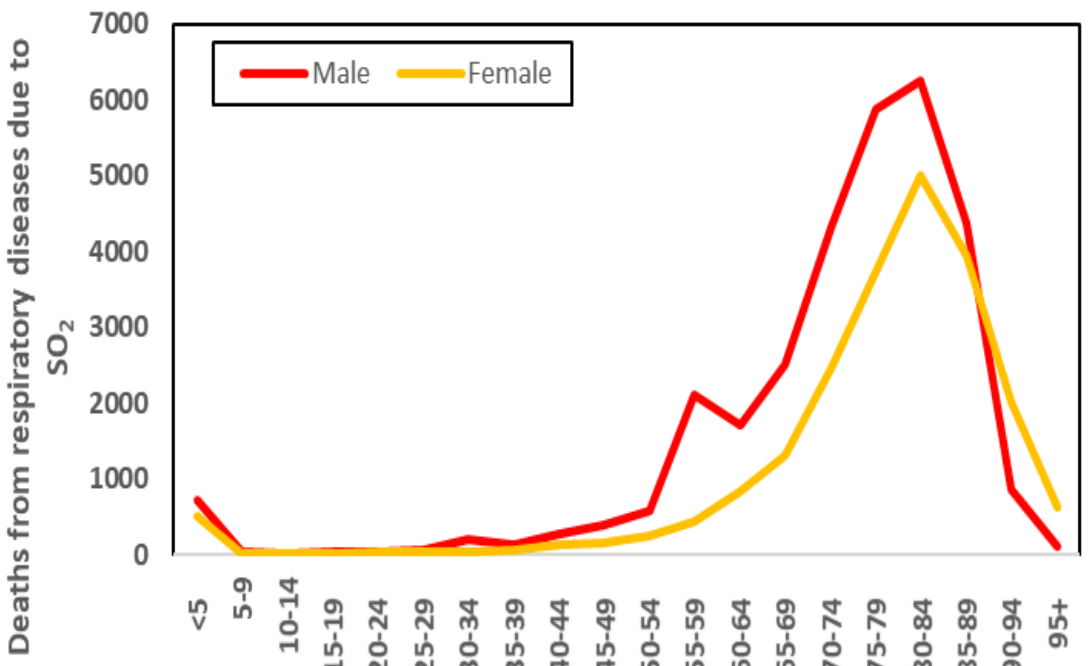

(c)

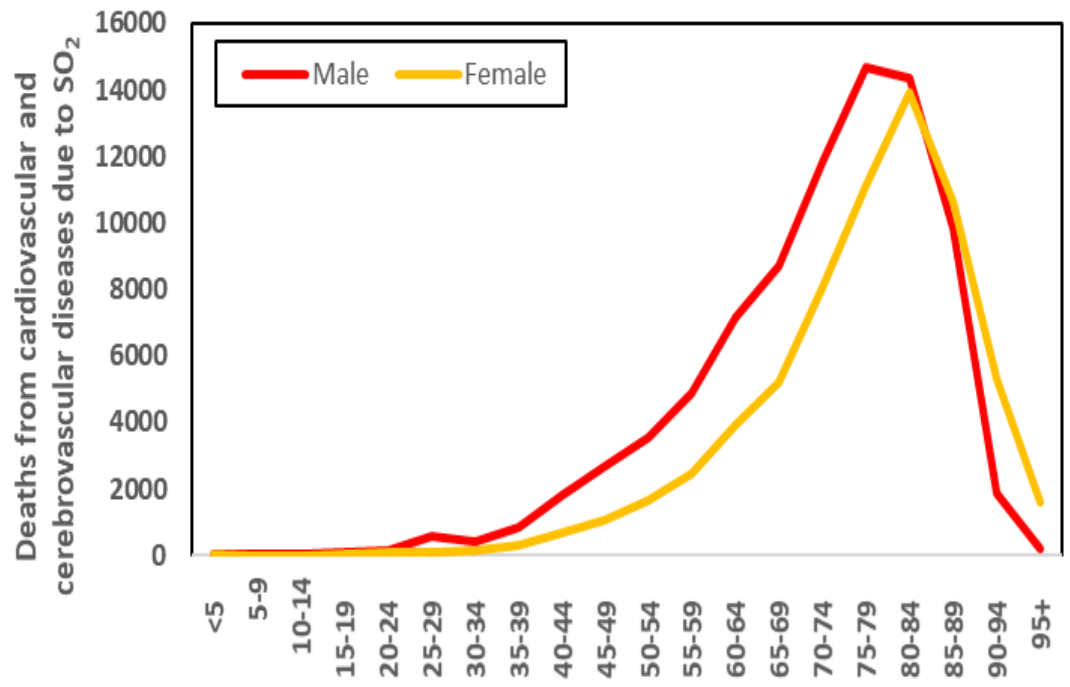

(d)

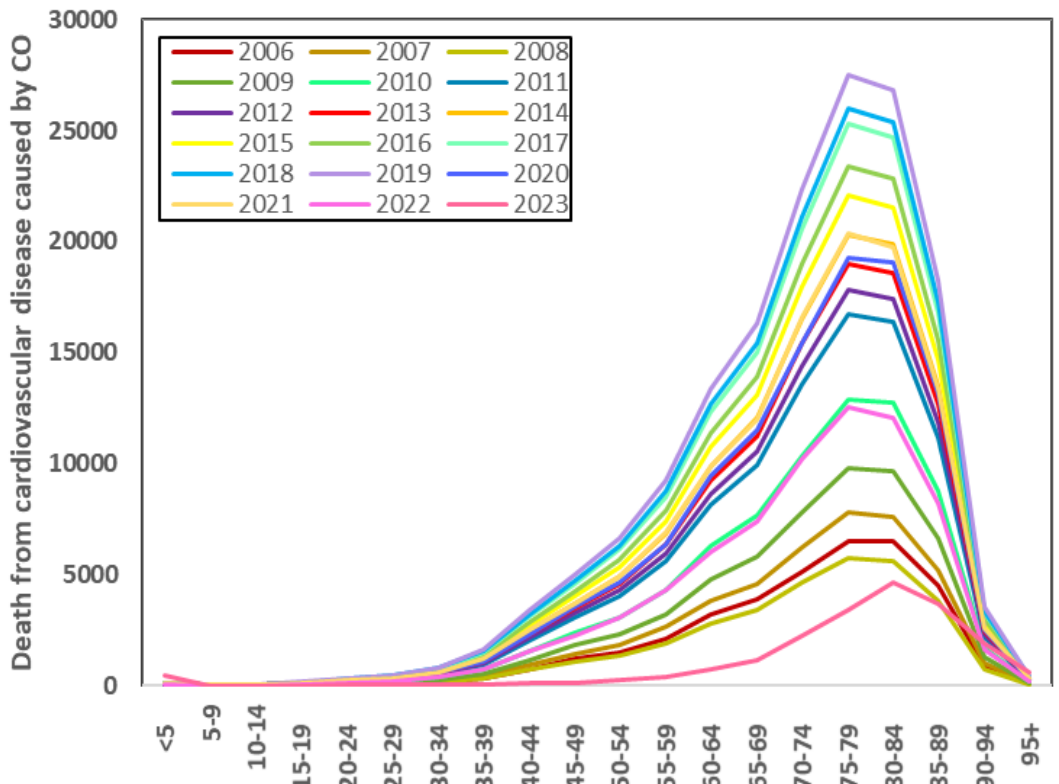
Death from cardiovascular disease caused by CO
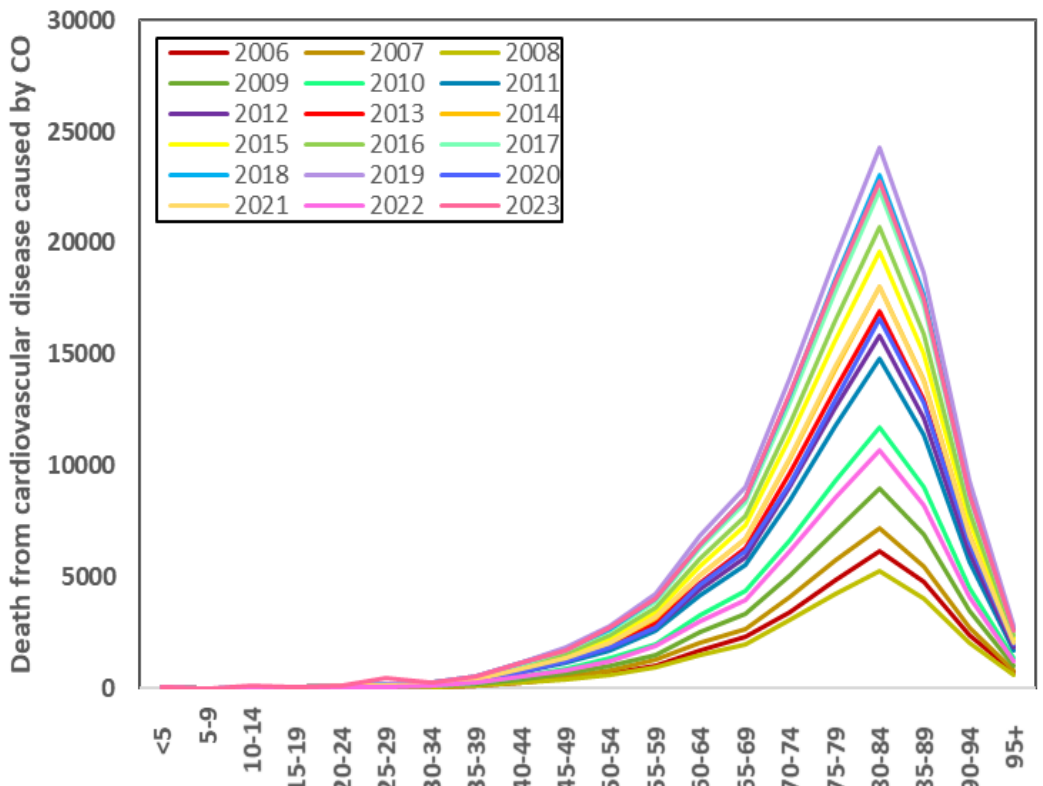
Death from cardiovascular disease caused by CO

(e) (f)

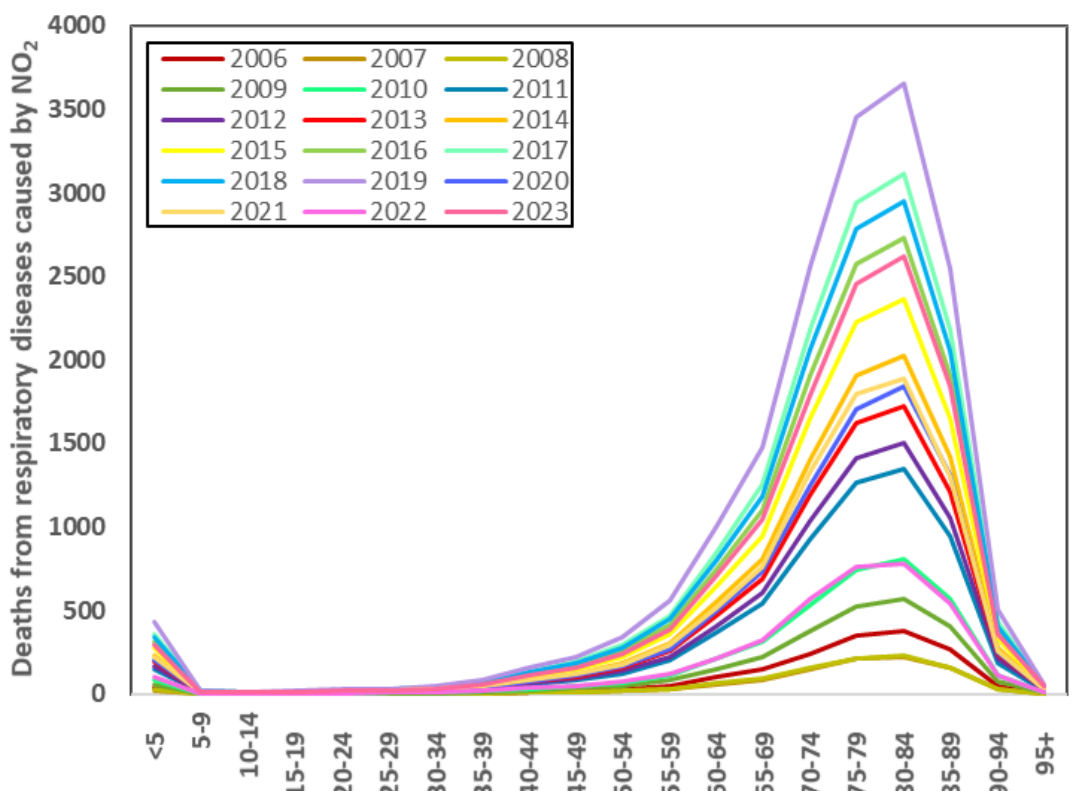
Deaths from respiratory diseases caused by NO_2
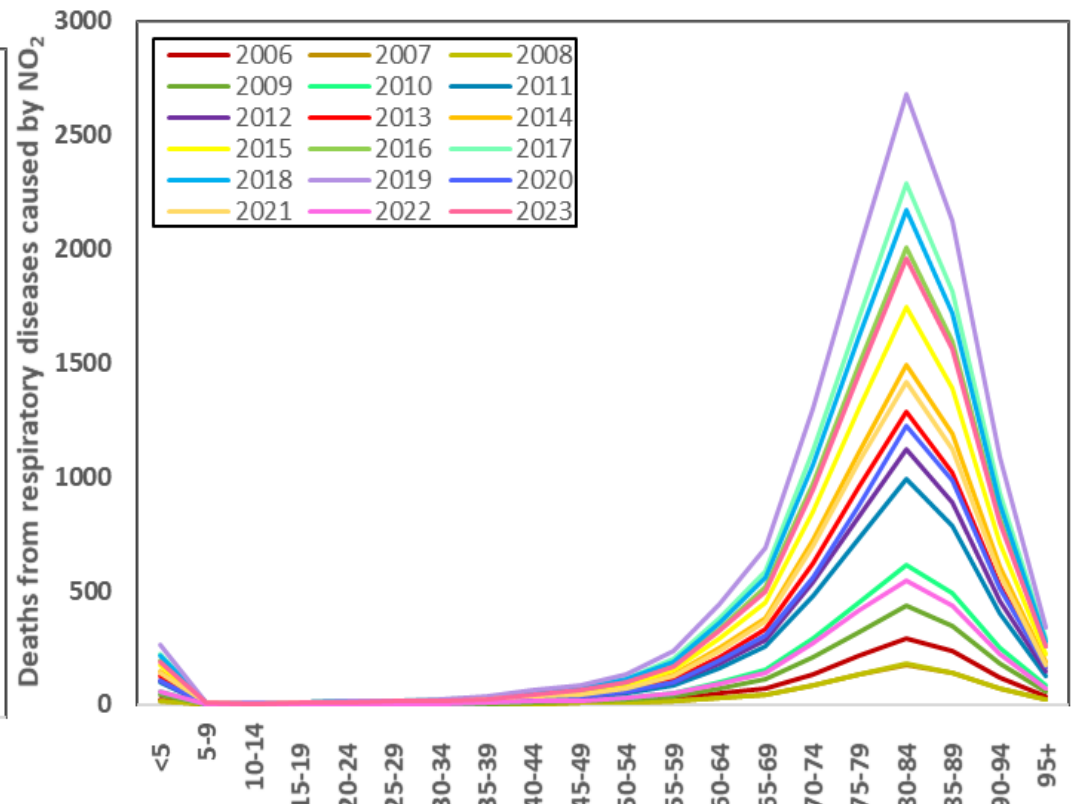
Deaths from respiratory diseases caused by NO_2

(g) (h)

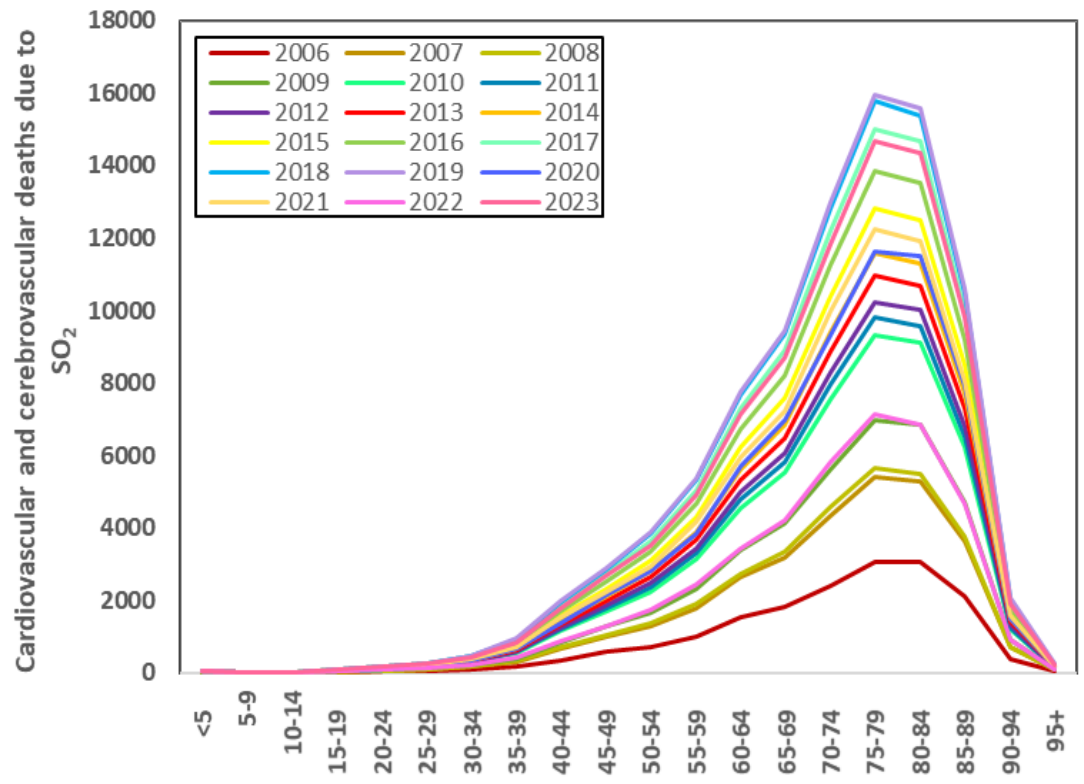
Cardiovascular and cerebrovascular deaths due to SO_2
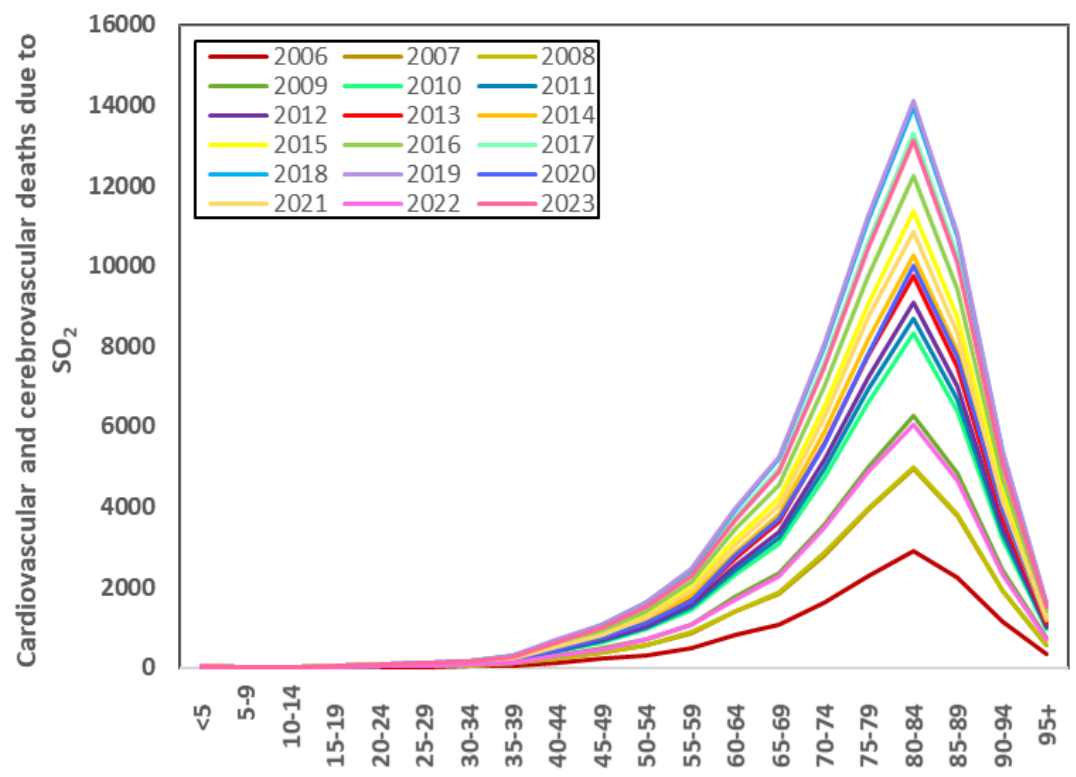
Cardiovascular and cerebrovascular deaths due to SO_2

(i) (j)

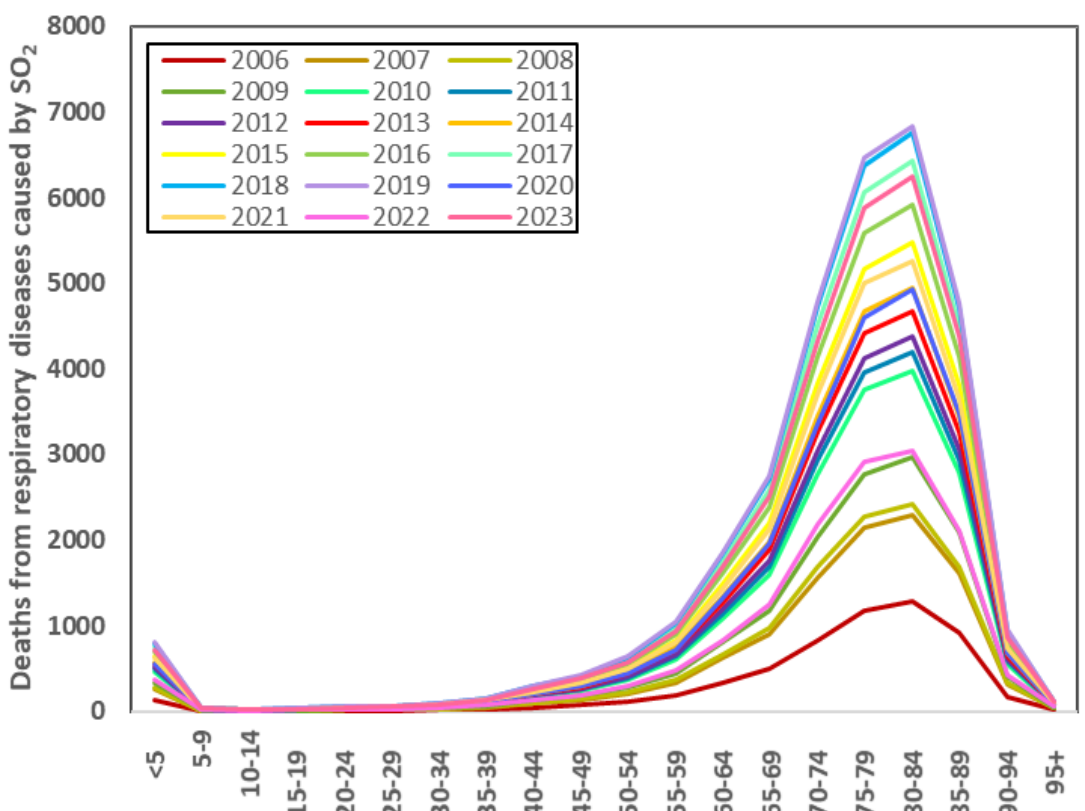
Deaths from respiratory diseases caused by SO_2
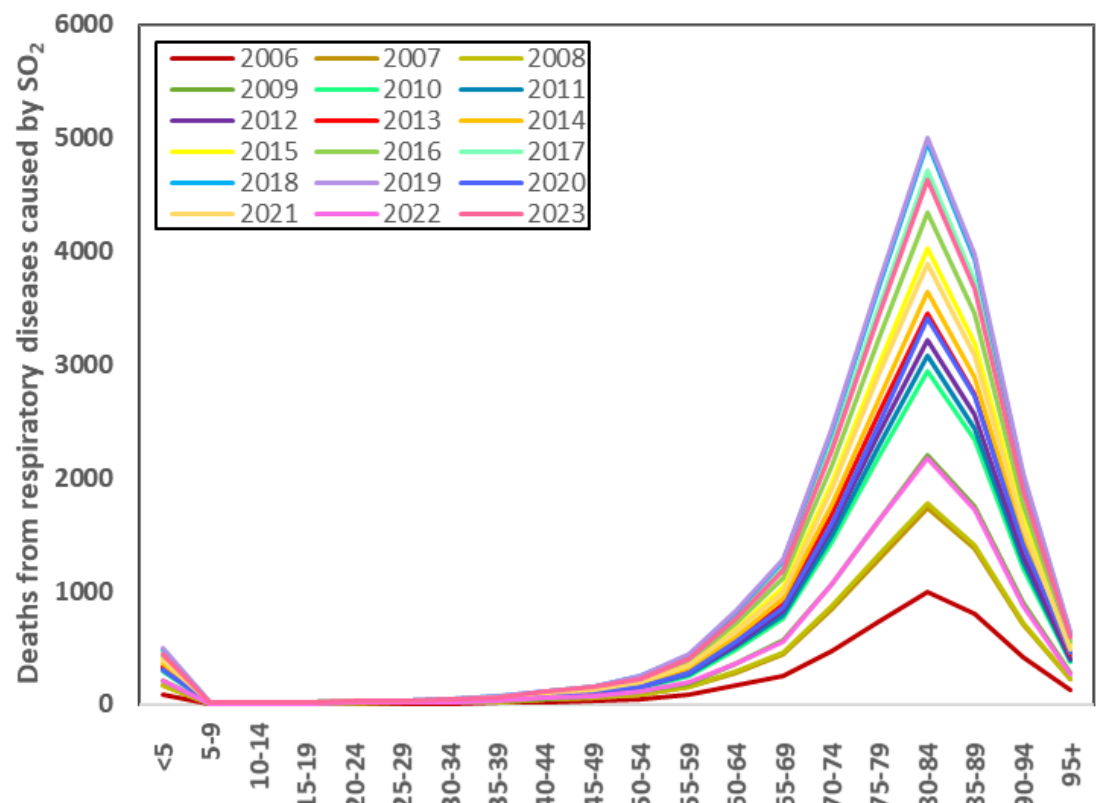
Deaths from respiratory diseases caused by SO_2

(k) (l)

Figure S2 Deaths due to CO, NO_2, SO_2 by age group from 2006 to 2023. (a) Number of deaths in men and women from cardiovascular disease due to CO; (b) Number of deaths in men and women from respiratory disease due to NO_2; (c) Number of deaths in men and women from respiratory disease due to SO_2; (d) Number of deaths in men and women from cardiovascular disease due to SO_2.; (e) Number of deaths from cardiovascular disease caused by CO in men of all ages from 2006 to 2023; (f) Number of deaths from cardiovascular disease caused by CO in women, (g) Number of deaths from respiratory disease caused by NO_2 in men at all ages from 2006 to 2023, (h) Number of deaths from respiratory disease caused by NO_2 in women, and (i) Number of deaths from cardiovascular disease caused by SO_2 in men of all ages from 2006 to 2023, (j) Number of deaths from cardiovascular disease caused by SO_2 in women, (k) Number of male deaths from respiratory diseases caused by SO_2 at all ages from 2006 to 2023; (l) Number of female deaths from respiratory diseases caused by SO_2.
